# Simulation Study on the Mechanical Properties of Fuzz Buttons

**DOI:** 10.3390/ma19132927

**Published:** 2026-07-07

**Authors:** Xiuping Dong, Zhongping Zhang, Mingji Huang

**Affiliations:** 1 School of Computer and Artificial Intelligence, University of Technology and Business Beijing, Beijing 100048, China; dongxp@th.btbu.edu.cn (X.D.); 2330601047@st.btbu.edu.cn (Z.Z.); 2 School of Mechanical Engineering, University of Science and Technology Beijing, Beijing 100083, China

**Keywords:** fuzz button, microstructure model, simulation method, mechanical performance, damping and energy dissipation

## Abstract

Fuzz buttons are formed by interweaving and compacting fine metallic wires, resulting in a highly porous architecture with complex internal contact interactions. Their compressive behavior is governed by the evolution of wire–wire contacts, frictional sliding, local bending, and plastic deformation, which cannot be adequately captured by conventional homogenized models. To address this limitation, a process-informed finite element modeling approach based on virtual fabrication is proposed. First, the spatial trajectories of 24 beryllium copper wires are generated using a parametric three-dimensional weaving algorithm and smoothed by cubic spline interpolation to obtain continuous wire centerlines. The resulting preform is then virtually compacted to reconstruct the densified wire network and its contact topology. The model employs a globally controlled solid-element mesh, a penalty-based general contact algorithm, a Coulomb friction model, and an explicit quasi-static solution scheme. The size-dependent plastic response of the fine wires is further incorporated through a Nix–Gao-based correction to the constitutive relation. The model is validated against quasi-static compression experiments at compressive strains of 15%, 20%, and 25%. The relative errors in the predicted peak forces are 2.12%, 5.65%, and 6.81%, respectively, while the corresponding coefficients of determination for the force–displacement curves are 0.984, 0.970, and 0.973. The model successfully reproduces the nonlinear loading–unloading response and hysteretic energy dissipation over the investigated strain range. The proposed approach provides a physically grounded numerical framework for predicting the compressive behavior of fuzz buttons and investigating the mesoscopic mechanics of complex interwoven wire networks.

## 1. Introduction

**Figure 1:**
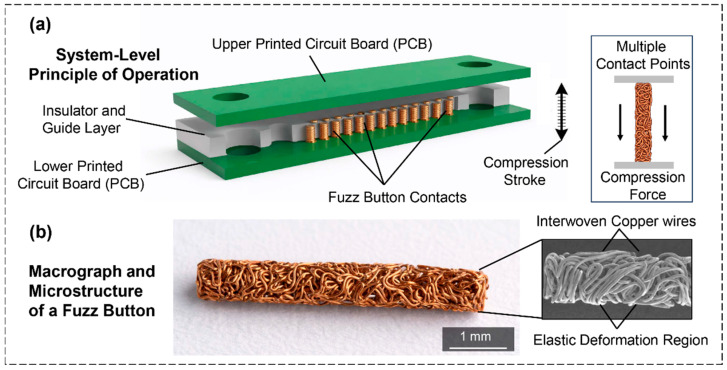
Schematic illustration of fuzz button vertical interconnection and high-magnification macroscopic image of the physical specimen: (**a**) System-Level Principle of Operation; (**b**) Macrograph and Microstructure of a Fuzz Button.

With the continuous evolution of electronic packaging technologies toward miniaturization, high-density integration, and high reliability, micro-interconnection structures are increasingly subjected to severe electro-thermo-mechanical multiphysics coupling challenges under complex service environments [1,2,3]. As a novel solderless vertical interconnection unit, the fuzz button demonstrates significant application potential in aerospace electronics, high-density PCB assembly, automotive electronics, and high-frequency communication modules due to its low profile, separable interconnection capability, short transmission path, and superior comprehensive electro-thermo-mechanical performance [4,5,6,7]. The principle of vertical interconnection is illustrated in Figure 1. Compared with conventional radio-frequency connectors and flip-chip bonding technologies, fuzz buttons form conductive pathways through the entanglement of internal metallic wires, thereby effectively shortening signal transmission distances and reducing reliability risks associated with solder joint fatigue failure and interface misalignment [8,9,10]. Therefore, establishing a high-fidelity model capable of accurately representing the internal mesoscopic structure of fuzz buttons and investigating their mechanical behavior on this basis are of considerable theoretical significance and engineering value for the reliable application of such interconnection structures.

Existing studies on the mechanical and electrical properties of fuzz buttons have mainly focused on experimental characterization and theoretical modeling. In terms of experimental investigations, Harris et al. [11] systematically evaluated the force–displacement behavior and contact resistance evolution of fuzz button contacts fabricated from different materials during compression. Their results indicated that the electrical resistance decreased rapidly with increasing compression displacement and gradually stabilized; however, the inherent randomness of the wire entanglement process resulted in significant dispersion in the mechanical responses among different specimens. Lv et al. [12] further investigated the rebound behavior of fuzz buttons fabricated from different materials under cyclic compression conditions from the perspective of intrinsic wire properties, revealing the critical influence of material mechanical properties on rebound stability. These studies provided fundamental experimental support for material selection and mechanical performance evaluation of fuzz buttons.

At the theoretical modeling level, researchers have attempted to correlate the complex internal structure of fuzz buttons with their macroscopic mechanical responses using equivalent modeling approaches. Huang et al. [13] simplified the random metallic wire network into polyline beam elements and established a macroscopic constitutive model of the fuzz button based on an equivalent spring network, thereby investigating the effects of wire diameter, elastic modulus, relative density, and geometric parameters on the overall compressive response. Zhu et al. [14] adopted an equivalent homogenization approach to establish a finite element model of a fuzz button connector assembly and analyzed load transfer, stress distribution, and structural response under impact loading conditions during compression. In addition, Zhang et al. [15] developed electrical simulation models with different hierarchical levels based on the actual fuzz button configuration and verified their effectiveness in predicting electrical performance. Lall et al. [16] established a numerical model to investigate contact surface wear behavior, while Zhu et al. [17] compared the rebound performance of fuzz buttons fabricated from different materials through simulation analysis. Zhang et al. [18] proposed a finite element modeling method based on digital manufacturing technology, enabling full-process simulation from wire-entangled preform generation to compression molding.

Beyond contact effects in wire mesh buttons, recent studies on architected lattices and mechanical metamaterials have emphasized the importance of geometry-governed mechanical behavior [19]. In lattice metamaterials, geometric features such as unit-cell topology can control the overall stiffness, deformation mode, buckling stability, and energy dissipation. Similarly, the compressive response of wire mesh buttons depends not only on the constitutive properties of beryllium copper wires, but also on the mesoscopic arrangement of the interwoven wire network. This analogy suggests that the internal structure of wire mesh buttons should be explicitly modeled rather than described solely by homogenized properties, particularly when nonlinear contact evolution, hysteresis, and energy dissipation are involved.

Although these studies have advanced the structural design and engineering application of fuzz buttons from various perspectives, equivalent homogenization approaches and simplified idealized geometric models remain insufficient for accurately characterizing the inherently porous, disordered, and interwoven mesoscopic topological features of fuzz buttons. Significant limitations still exist in describing critical mechanical behaviors, including contact nonlinearity, large deformation, and local plastic evolution.

Although preliminary studies have explored modeling approaches based on realistic mesoscopic geometries, limited research has been conducted on finite element solution strategies for such extremely complex porous network structures. The interior of a fuzz button contains a massive number of randomly distributed spatial metallic wire contact pairs, which undergo strongly nonlinear evolution from point contact and relative sliding to localized severe extrusion during loading and unloading processes. Existing simulation studies have primarily focused on the output of macroscopic responses or idealized structural representations, whereas investigations on full-cycle numerical simulation strategies encompassing preprocessing, solver configuration, and postprocessing remain insufficient. The oversimplified treatment of the underlying solution mechanisms often leads to computational bottlenecks under large compression strokes, including contact penetration, mesh distortion, and convergence difficulties.

To address these issues, this study proposes a finite element modeling method for wire mesh buttons based on virtual fabrication. The method abstracts the actual manufacturing process, including wire winding and compression densification. An initial porous preform is first generated using a three-dimensional weaving trajectory, followed by virtual compression to obtain the densified wire mesh button model. In the finite element analysis, a global mesh control strategy, a penalty-based general contact algorithm, and a Coulomb friction model are employed to describe the complex contact and sliding behavior between wires. Quantitative evaluation methods are further established for the force–displacement response, hysteretic energy dissipation, loss factor, and average stiffness. The model is validated through quasi-static compression experiments. This approach enables the analysis of nonlinear contact response and hysteretic energy dissipation during compression, providing a feasible numerical framework for structural modeling and mechanical performance prediction of wire mesh buttons.

## 2. Virtual Fabrication of Fuzz Buttons

### 2.1. Principle of Preform Trajectory Generation

**Figure 2:**
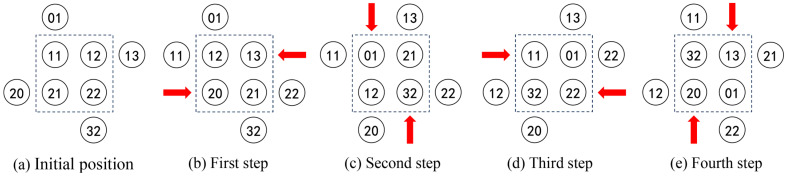
Process Diagram of the Four-Step Braiding Method for 3D Models.

The internal structure of a fuzz button contact is formed by the mutual entanglement, stacking, and subsequent compression molding of fine metallic wires in three-dimensional space, resulting in a mesoscopic structure characterized by significant porosity, randomness, and complex contact features [20,21,22]. Owing to the disordered arrangement and complex geometric morphology of metallic wires inside actual fuzz buttons, it is difficult to directly establish a finite element model with realistic topological characteristics. Therefore, combined with the actual fabrication process of fuzz buttons, a virtual fabrication strategy was adopted to digitally reconstruct both the preform structure and the forming process. In practical manufacturing, the fabrication of fuzz buttons generally includes metallic wire pretreatment, wire entanglement forming, compression molding, and subsequent heat treatment processes [23]. The essence of the preform structure lies in the continuous spatial braiding and interlaced accumulation of metallic wires. Accordingly, a three-dimensional braiding “four-step method” was introduced to parametrically describe the trajectories of metallic wires. The core concept of this method is to arrange metallic wires spatially according to prescribed rules and impose periodic motions along the X-, Y-, and Z-directions [24,25,26]. A complete braiding cycle sequentially consists of row-wise movement, column-wise movement, reverse row-wise movement, and reverse column-wise movement, through which the metallic wires gradually form an integrated three-dimensional porous network structure. The principle of the three-dimensional braiding four-step method is illustrated in Figure 2.

The proposed method does not assume the internal solid morphology of the fuzz button in advance; rather, it derives the preform structure from the spatial motion of metallic wires, transforming the generation of the preform into a trajectory evolution problem. By adjusting parameters such as braiding step length, motion direction, and the number of cycles, the external dimensions, internal pore distribution, and overall density of the preform can be controlled, yielding a model with a clear geometric origin and parametrizable features suitable for subsequent finite element analysis.

The spatial motion of metallic wires during the braiding process is categorized into four fundamental behaviors—motion, direction change, fixation, and reset—to construct the central trajectory. These four behaviors act synergistically within a single braiding cycle to drive the continuous evolution of the wires. Table 1 summarizes the complete steps of one braiding cycle in the four-step method.

The motion behavior describes the continuous extension of the wire along a prescribed direction in space and constitutes the primary mechanism for forming the basic preform skeleton. This behavior is determined jointly by the direction of motion and the step length. Motion in the X and Y directions governs the planar distribution and winding pattern of the wire, while motion in the Z direction controls the interlayer stacking along the thickness. By setting the step lengths in each direction, preliminary control over the preform dimensions and porosity can be achieved.

The direction-change behavior is activated when a metallic wire reaches a preset boundary, redistributing its motion direction so that planar extension ceases and interlayer transitions along the thickness direction are completed. This behavior ensures that the trajectories form a uniform and symmetric distribution in space, preventing local structural looseness and providing a stable initial geometric basis for subsequent compression molding.

After the local trajectory of a wire is generated, fixation behavior is applied to enhance the overall stability of the preform structure by establishing connections between adjacent trajectories. Fixation includes two forms: intra-layer adjacent fixation and interlayer connection fixation. The former creates a relatively stable planar support within each layer, while the latter enables load transfer between layers. Together, they construct a complete three-dimensional network topology, transforming the preform from a simple collection of trajectories into an integrated spatially braided structure.

Upon completing a braiding cycle, the wire trajectories are returned to a new initial state for the next cycle, a process defined as the reset behavior. This behavior ensures the periodicity of trajectory evolution, allowing a single metallic wire to continuously extend according to the same rules while imparting a macroscopically uniform, repetitive stacking feature to the preform structure. By adjusting the number of cycles and the Z-direction step length, the overall height and internal layer distribution of the preform can be further controlled. Through the continuous interaction of these four behaviors, discrete spatial trajectory points for each metallic wire in the fuzz button preform can be generated.

### 2.2. Model Generation Parameters

The model was generated using the macroscopic dimensions and material volume fraction of the target wire mesh button as constraints, including the diameter (*D*), height (*H*), wire diameter (*d*), filling ratio (*f*), and porosity (*φ*). The compacted wire mesh button was assumed to be a cylindrical model, with its main parameters listed in Table 2.

The total volume of the formed specimen was determined from the geometric relationship of a cylinder:

(1) $$V_{FB}=\pi {\left(\frac{D}{2}\right)}^{2}H=\frac{\pi HD^{2}}{4}$$

(2) $$V_{wire}=V_{FB}f=\frac{\pi fHD^{2}}{4}$$

(3) $$L_{wire}=\frac{V_{wire}}{\frac{\pi d^{2}}{4}}=\frac{fHD^{2}}{d^{2}}$$

where $V_{FB}$ is the volume of the wire mesh button after compaction, $V_{wire}$ is the total volume of the metallic wires after compaction, and $L_{wire}$ is the total wire length. The total volume of metallic wires was determined by the filling ratio, and the total wire length was obtained from the wire volume and cross-sectional area. Therefore, the wire content in the model was consistent with that of the real specimen in terms of volume fraction.

During trajectory generation, the wire centerlines were defined by the dimensions of the core coding region, *m* and *n*, the motion step sizes in the X/Y directions, *A*, the motion step size in the *Z* direction, *B*, and the number of resets, $N_{reset}$. The optimized parameter set for generating the formed wire mesh button is listed in Table 3. The total number of wire trajectories in the model was determined by the initial coding scheme, expressed as $N_{all}=m^{2}+2m$.

These wire trajectories were not prescribed arbitrarily, but were generated continuously through four basic operations: movement, direction change, fixation, and reset. Cubic spline interpolation was then applied to smooth the discrete trajectory points, thereby avoiding unrealistic sharp corners that could affect the subsequent contact analysis. By adjusting *A*, *B*, and $N_{reset}$, the difference between the spline-fitted total wire length and the target wire length calculated from the filling ratio was minimized. This ensured that the model satisfied the target specimen parameters in terms of wire content, porosity, and preform dimensions.

### 2.3. Three-Dimensional Preform Modeling

**Figure 3:**
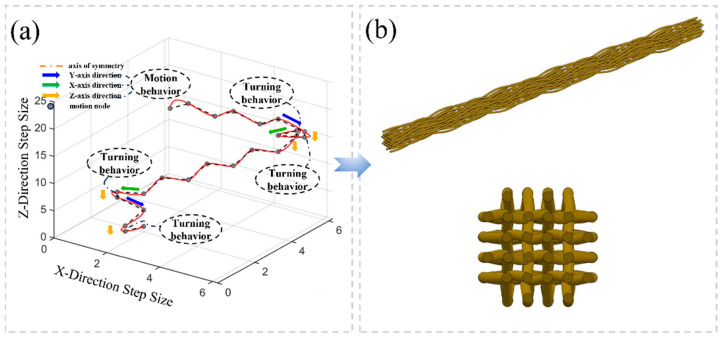
Generation process of fuzz button preform: (**a**) Preform trajectory coordinates; (**b**) Three-dimensional preform model.

Directly connecting the discrete trajectory points can easily produce geometric sharp corners and local discontinuities, which adversely affect the accuracy and convergence of subsequent contact analysis. To address this issue, cubic spline interpolation was employed to smoothly fit the trajectory of each metallic wire, yielding continuous and smooth spatial curves [27,28,29], as shown in Figure 3a. Based on this, the fitted trajectory coordinate data were imported into the SolidWorks 2022 environment via the SolidWorks API interface. In SolidWorks, the discrete trajectory points were connected using smooth curves to create the preform sketches. Subsequently, each wire trajectory curve was swept with the metallic wire radius as the sweeping parameter, producing the three-dimensional preform model of the fuzz button, as illustrated in Figure 3b. The specifications of the fuzz button preform are listed in Table 4.

### 2.4. Three-Dimensional Modeling of Fuzz Buttons

The constructed preform model reflects only the initial configuration of the metallic wires during the braiding stage and cannot be directly used as a finite element model of the fuzz button in its service state. In the actual fabrication process, the preform undergoes compression molding, experiencing significant axial compression, radial expansion, and internal contact reorganization under external forces. Therefore, it is necessary to further geometrically refine the preform and reconstruct its contact state through a virtual compression process.

Virtual compression modeling first requires the construction of a stamping die. The target diameter of the fuzz button was used as the design basis for the inner diameter of the cylindrical die cavity, while the die wall thickness and outer diameter were determined by considering machining accuracy and structural rigidity requirements [30,31]. The die height was modeled in segments according to the preform height and punch stroke, and the bottom structure was refined to finalize the three-dimensional cylindrical die model.

**Figure 4:**
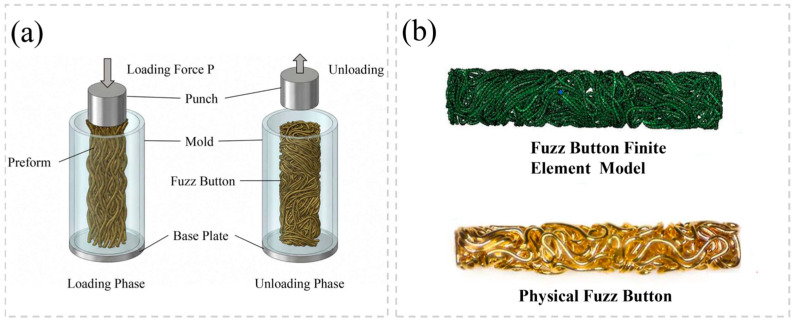
Numerical modeling of the fuzz button forming process: (**a**) Schematic of compression; (**b**) Comparison between fuzz button model and physical specimen.

Based on this, an assembly of punch–preform–die was established in Abaqus. The metallic wires of the preform were defined as deformable bodies, while the punch and die were treated as rigid boundary components. Axial compression of the preform was driven by displacement-controlled motion of the punch. Following a prescribed time–displacement history, the loading, holding, and unloading stages were sequentially simulated, representing the entire process of the fuzz button transitioning from a loose preform to a densified molded component, as illustrated in Figure 4a. After compression, elastic recovery of the preform produced a certain amount of springback, resulting in a final geometry that closely matches the physical fuzz button in both external dimensions and internal structural features, as shown in Figure 4b.

## 3. Fuzz Button Finite Element Simulation Method

### 3.1. Finite Element Preprocessing

The three-dimensional mesoscopic model of the fuzz button was imported, and an assembly of punch–fuzz button–die was established in Abaqus. The fuzz button consists of multiple solid metallic wires, retaining the realistic spatial entanglement and initial contact topology among the wires. The punch is positioned above the fuzz button, while the die is located below, together forming the basic assembly for compression analysis. Considering that the primary deformation of the fuzz button during compression is concentrated within the internal wire network and that the punch and die possess high rigidity with negligible deformation, the punch and die were simplified as rigid bodies, while only the fuzz button was defined as a deformable body. This treatment reduces the model’s degrees of freedom, improves computational efficiency, and focuses the analysis on the stress and deformation behavior of the fuzz button’s complex internal structure while maintaining simulation accuracy.

Wire mesh buttons are commonly made of beryllium copper alloy. In this study, beryllium copper alloy was used as the reference material for finite element modeling, and the corresponding material parameters were assigned based on supplier technical datasheets and the Chinese national standard GB/T 33220-2016 [32]. The material parameters of the wires included the elastic modulus, Poisson’s ratio, and density, which were set as 128 GPa, 0.3, and 8.25 g/cm^3^, respectively. The material properties used in the model are listed in Table 5.

During compression, the metallic wires within the fuzz button undergo not only bending-induced yielding and contact extrusion but also irreversible plastic deformation. When the wire diameter is reduced to the order of tens of micrometers, the number of internal grains decreases significantly, and conventional macroscopic continuum mechanics models fail to accurately capture the mechanical response. In this regime, the material exhibits a pronounced size effect in which the yield strength increases as the characteristic dimension decreases (“smaller is stronger”) [33,34,35]. To reasonably incorporate such microscale effects into the simulation, a correction method based on strain gradient plasticity theory was adopted [36,37,38]. Considering that the forming and compression processes of fuzz buttons involve numerous microscale bending regions, directly solving higher-order strain gradient equations would substantially increase computational cost and lead to convergence difficulties. Therefore, an equivalent strategy was employed in which the material constitutive curve is directly corrected, embedding the microscale effects into the finite element model without additional computational burden and thereby improving the prediction accuracy of forming springback.

The plastic strain data were corrected according to the Nix–Gao strain gradient plasticity theory [39], using the following Equations (4) and (5):

(4) $$l^{*}=\frac{M^{2}\alpha^{2}G^{2}b}{\sigma_{0}^{2}}$$

(5) $$K=\sqrt{1+\frac{l^{*}}{R_{bend}}}$$

where *M* is the Taylor factor, *α* is a material-dependent empirical constant, *G* is the shear modulus, *b* is the magnitude of the Burgers vector, $\sigma_{0}$ is the flow stress in the macroscopic state without size effects, $l^{*}$ is the characteristic intrinsic length, $R_{bend}$ is the characteristic bending radius, and *K* is the stress correction amplification factor.

**Figure 5:**
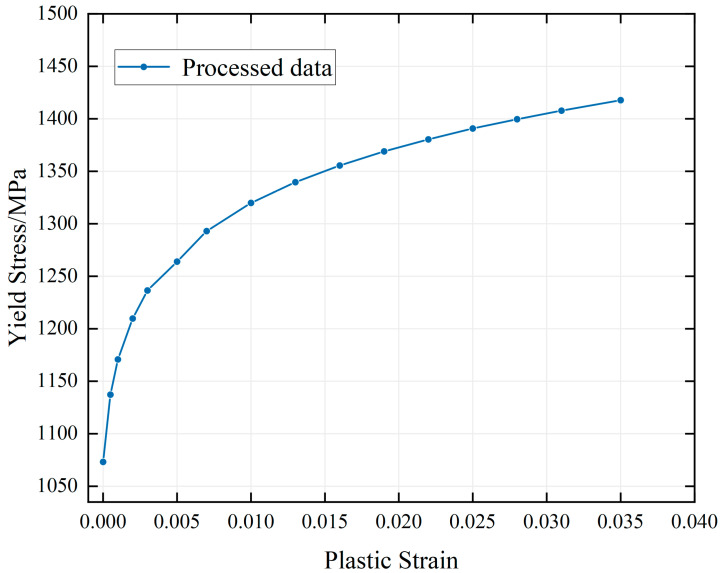
Corrected stress–strain curve.

For beryllium copper alloy, the parameters used in the Nix–Gao correction were selected based on the characteristic material length scale and the geometric features of the wire. The intrinsic characteristic length $l^{*}$ was set to 0.003 mm. The characteristic bending radius $R_{bend}$ was estimated from the wire geometry. Given a wire diameter of 0.08 mm, the local bending radius in highly curved contact regions was approximated as half of the wire diameter, i.e., $R_{bend}$ = 0.04 mm. Substituting these values into the Nix–Gao-based correction equation gives a stress amplification factor of K = 1.0368, indicating that the size-effect correction increases the yield stress by approximately 3.68%. Therefore, under the present wire diameter, this correction has a limited influence on the overall mechanical response. Nevertheless, it was retained to provide a more physically consistent description of microscale plastic deformation in the wires. The corrected yield stress–plastic strain curve is presented in Figure 5.

A comparative analysis was conducted with and without the Nix–Gao correction. Except for the plastic constitutive curve, the two models used the same geometry, mesh, contact parameters, boundary conditions, and loading path. The uncorrected model adopted the macroscopic yield stress–plastic strain curve of beryllium copper alloy. In the corrected model, the yield stress was uniformly increased by 3.68% according to the stress amplification factor calculated from the Nix–Gao theory (K = 1.0368), and the corrected yield stress–plastic strain curve was implemented in the Abaqus material module.

Table 6 compares the simulation results obtained with and without the Nix–Gao correction against the experimental result at a compressive strain of 20%. After applying the correction, the simulated peak reaction force increased from 0.7798 N to 0.7936 N, reducing the relative error with respect to the experimental value of 0.8411 N from 7.29% to 5.65%. Meanwhile, the coefficient of determination of the force–displacement curve increased from 0.965 to 0.970. These results indicate that the Nix–Gao-based correction provides a modest improvement in the predicted compressive response.

During compression, the metallic wires within the fuzz button undergo not only overall bending deformation but also localized contact extrusion, plastic evolution, and multiple random contact points. Therefore, solid elements suitable for complex contact and large deformation problems are required for discretization. In this study, the metallic wires were meshed using eight-node linear reduced integration three-dimensional solid elements (C3D8R) in Abaqus. These elements employ a reduced integration scheme that balances computational efficiency with solution accuracy, effectively lowering the computational cost of large-scale contact analysis while demonstrating good compatibility with explicit dynamic solvers, making them suitable for handling the highly nonlinear behavior of the fuzz button mesoscopic model [40]. The punch and die were simplified as rigid bodies with negligible deformation; thus, they could be discretized using coarser meshes or analytical rigid body representations, as they are not the primary focus of deformation analysis.

A globally controlled meshing strategy was adopted for the wire network. To evaluate the suitability of the selected mesh size, a mesh convergence study was performed, as summarized in Table 7. The relative error in the peak reaction force decreased with mesh refinement. When the mesh size was reduced from 0.052 mm to 0.040 mm, the peak reaction force changed from 0.7936 N to 0.8054 N, with a relative difference of only 1.47%. Considering both computational accuracy and efficiency, a mesh size of 0.052 mm was used in the subsequent simulations. This mesh setting ensured sufficient numerical accuracy while controlling the model size and avoiding element penetration or collapse during compaction. The element types and mesh numbers of each component are summarized in Table 8, where the punch was meshed using C3D8R elements and the die was meshed using C3D10M elements.

During compression, the fuzz button involves complex multipoint contact interactions, relative sliding, and localized extrusion not only among metallic wires but also between the wires and the punch and die. Therefore, the proper definition of contact relationships is critical for finite element simulation. Accordingly, contact pairs were established for wire–wire, wire–punch, and wire–die interactions to simulate the dynamic evolution of contact states during compression and unloading.

In the mesoscopic structure of wire mesh buttons, increasing load reduces the internal pores and promotes contact, sliding, and friction between adjacent wires. During unloading, the pores reopen and some contact pairs separate. Therefore, the wire–wire interaction should be treated as frictional contact. Under cyclic loading, repeated contact, separation, and sliding occur within the wire network. In this case, the penalty contact formulation is more suitable than a hard constraint formulation because it generally provides better numerical convergence.

The normal pressure–overclosure response was defined as hard contact, allowing separation after contact. The tangential contact behavior was described using a Coulomb friction model implemented with the penalty formulation. General contact in Abaqus/Explicit was employed to account for wire–wire, wire–punch, and wire–die interactions, thereby allowing the contact pairs to evolve automatically during loading and unloading.

For three-dimensional contact problems, the contact interfaces exhibit strong nonlinearity, and the governing equations consist of the coupling between equilibrium equations and contact constraints. The virtual work equation with contact constraints can be expressed as follows [41]:

(6) $$G\left(u_{t},u_{\delta }\right)-\int_{\Gamma_{c}}P_{c}^{t}\times u_{\delta }d\Gamma_{c}^{t}=0$$

where $G\left(u_{t},u_{\delta }\right)=\int_{\Omega }\sigma_{t}:grad\left(u_{\delta }\right)d\Omega_{t}-\int_{\Omega }F_{t}\times u_{\delta }d\Omega_{t}-\int_{\Gamma_{s}}T_{t}\times u_{\delta }d\Gamma_{s}^{t}$, $G\left(u_{t},u_{\delta }\right)$ is the virtual work functional, $u_{t}$ is the actual displacement, and $u_{\delta }$ is the virtual displacement, $\Gamma_{s}^{t}$ denotes the boundary condition subjected to external forces, and $\Gamma_{c}^{t}$ represents the boundary of the potential contact region. “Grad” denotes the gradient operator, and $\Omega_{t}$ represents the contact system, while $\sigma_{t}$, $F_{t}$, $T_{t}$, and $P_{c}^{t}$ denote the stress, body force, surface traction, and contact force, respectively. Consistent with the equilibrium conditions on the stress boundary, the contact penetration at the contact point $X\in \Gamma_{c}$ can be determined through Equation (6). On this basis, the relationship between the contact force and the contact displacement increment was established by combining the penalty function method with non-classical friction theory.

The nodal contact penetration at each load step can be determined using Equation (6). On this basis, the relationship between the contact force and the contact displacement increment was established by incorporating the penalty function and non-classical friction theory:

(7) $$dP_{c}=E_{ct}dg_{c}$$

(8) $$E_{ct}=\left[\begin{array}{ccc} \varepsilon_{T}\left(1-\omega \frac{P_{T1}^{2}}{{\left|P_{T}\right|}^{2}}\right) & -\omega \varepsilon_{T}\frac{P_{T1}P_{T2}}{{\left|P_{T}\right|}^{2}} & \omega \mu \varepsilon_{N}\frac{P_{T1}}{\left|P_{T}\right|}\\ -\omega \varepsilon_{T}\frac{P_{T1}P_{T2}}{{\left|P_{T}\right|}^{2}} & \varepsilon_{T}\left(1-\omega \frac{P_{T2}^{2}}{{\left|P_{T}\right|}^{2}}\right) & \omega \mu \varepsilon_{N}\frac{P_{T2}}{\left|P_{T}\right|}\\ 0 & 0 & \varepsilon_{N} \end{array}\right]$$

where $dP_{c}$ is the contact force increment vector, $dg_{c}$ is the contact displacement increment vector, $E_{ct}$ is the tangent stiffness matrix of the contact element, $\varepsilon_{T}$ is the tangential contact stiffness, $\varepsilon_{N}$ is the normal contact stiffness, $P_{T1}$ and $P_{T2}$ are the tangential contact force components, $P_{T}$ is the magnitude of the resultant tangential contact force, μ is the nonclassical friction parameter, and ω is the contact state factor. Specifically, ω = 0 corresponds to sticking contact, whereas ω = 1 represents the sliding state.

A sensitivity analysis was performed to determine an appropriate penalty contact-stiffness scale factor, and the results are summarized in Table 9. As the penalty stiffness scale factor increased, the contact penetration gradually decreased, indicating improved accuracy of contact enforcement. However, an excessively large stiffness scale factor may reduce the stable time increment, introduce high-frequency numerical oscillations, and substantially increase the computational cost in the explicit analysis. Conversely, an excessively small stiffness scale factor may lead to excessive contact penetration and an inaccurate representation of wire–wire contact interactions. Therefore, the penalty stiffness scale factor should be selected by balancing contact accuracy, numerical stability, and computational efficiency. Based on the sensitivity results, a penalty stiffness scale factor of 0.8 was adopted in the subsequent simulations.

The friction coefficient was further varied to evaluate its effect on the mechanical response of the wire mesh button. Only the tangential friction coefficient for wire–wire contact was changed, with values of 0.10, 0.12, 0.15, 0.20, and 0.25 considered. For each case, five loading–unloading cycles were simulated at a compressive strain of 0.20. The hysteresis loops were extracted to calculate the peak force, dissipated energy ${\bar{W}}_{load}$, and energy dissipation ratio $\bar{\lambda }$. The energy dissipation ratio was defined as $\bar{\lambda }$ = $({\bar{W}}_{d}/{\bar{W}}_{load})\times 100\%$, where ${\bar{W}}_{load}$ is the work done by the external force during loading and ${\bar{W}}_{d}$ is the energy dissipated during one complete loading–unloading cycle. The results are summarized in Table 10.

As the friction coefficient increased from 0.10 to 0.20, the frictional work generated by relative sliding between contacting wires increased, and the average dissipated energy rose from 5.1177 × 10^−5^ J to 6.3383 × 10^−5^ J, corresponding to an increase of approximately 23.85%. When the friction coefficient was further increased to 0.25, relative sliding between some contacting wires was suppressed, and the structure carried the load more through elastic deformation and local buckling. As a result, the average dissipated energy slightly decreased to 6.1742 × 10^−5^ J.

The average energy dissipation ratio generally increased with the friction coefficient and gradually approached saturation when *μ* > 0.20. Overall, the friction coefficient had a pronounced effect on hysteretic energy dissipation and rebound behavior, whereas its influence on the peak reaction force and average stiffness was relatively limited. This suggests that the load-bearing response of the model is not overly sensitive to the friction coefficient.

Based on the surface characteristics of beryllium copper alloy under dry, unlubricated conditions, the friction coefficient was set to 0.12. This value is consistent with the sensitivity analysis, supporting the rationality of the selected model parameter. This value determines the critical shear-stress threshold for the transition between sticking and sliding states in the contact stiffness matrix *E_ct_* in Equation (8). It can be inferred from the matrix structure of Equation (8) that the switching of the contact state exhibits pronounced nonlinear characteristics. For the large number of contact pairs inside the fuzz button, directly imposing contact constraints using the Lagrange multiplier method would lead to a sharp increase in the dimensionality of the system stiffness matrix and would readily induce saddle-point problems. By allowing a negligible amount of penetration, the penalty function method effectively ensures the numerical stability of large-scale random contact analyses and is therefore more suitable for the contact solution of the present model. The complete contact parameter settings are summarized in Table 11. The above contact definition can effectively capture the contact evolution characteristics of metallic wires inside the fuzz button during loading, progressing from localized point contact and sliding contact in the initial stage to compressive contact and localized sticking states in the middle and later stages.

### 3.2. Analysis Step and Solver Settings

During the compression forming of the fuzz button, the metallic wires continuously deform and displace under the applied load, causing the contact positions between the wires and the die surface, as well as the contact states among the wires, to evolve continuously throughout the loading process. This constitutes a typical contact nonlinearity problem [42]. When applied to such large-scale random contact problems, static implicit solution methods often suffer from convergence difficulties or local solution failures due to abrupt switching of contact states. Therefore, an explicit dynamic solver was employed to simulate the compression process. Explicit dynamics performs time integration based on the central difference method and does not require iterative solution of the tangent stiffness matrix, enabling stable treatment of highly nonlinear contact and complex large-deformation problems [43].

Owing to the high degree of nonlinearity of the model, direct use of explicit dynamic analysis would result in excessively small time increments and frequent increment reductions. Therefore, a quasi-static analysis strategy was adopted. To accurately characterize the quasi-static compression behavior, the loading rate must be strictly controlled and appropriate mass scaling must be introduced to ensure that the kinetic energy of the system remains below 5% of the internal energy, thereby eliminating the influence of inertial effects. The analysis step settings are listed in Table 12. The entire loading process was divided into loading and unloading stages according to the time–displacement amplitude curve, corresponding to the gradual compression of the fuzz button by the punch and the elastic recovery after punch removal, respectively. Smooth displacement amplitude functions were adopted in both stages to suppress dynamic oscillations induced by impact loading and to improve the accuracy of the quasi-static solution.

In terms of boundary conditions, the bottom of the die was fully constrained, with all translational and rotational degrees of freedom restricted. The punch was allowed to retain only the displacement degree of freedom along the compression direction (Z direction), while the remaining degrees of freedom were constrained. The bottom surface of the fuzz button was in contact with the die, whereas its upper surface was in contact with the punch, thereby establishing a complete axial compression path. The load was applied through a prescribed displacement history of the punch along the Z direction. The total loading displacement was set to 0.8 mm, and a staged displacement history was employed to complete the entire loading and unloading process, thereby approximating the actual compression conditions of the fuzz button. All simulations were performed using Abaqus/Explicit on a high-frequency cloud-based CPU computing platform. The final mesh model adopted in this study was solved in parallel using 16 CPU threads. For one complete loading–unloading simulation case, the wall-clock runtime was approximately 6–8 h. The actual runtime varied slightly among different cases because of the nonlinear evolution of wire–wire contact pairs during compression and unloading.

### 3.3. Post-Processing Method

**Figure 6:**
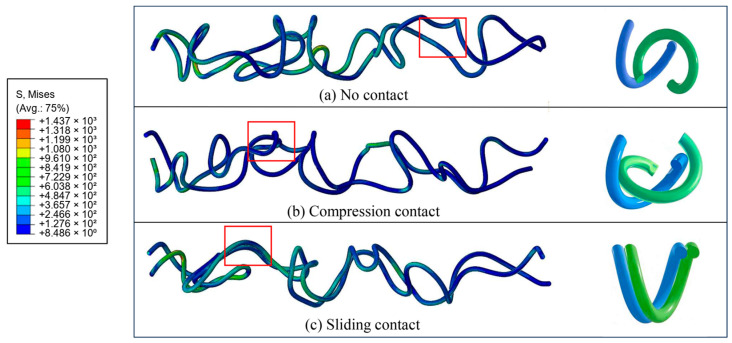
Evolution of contact state.

In the post-processing stage, typical local regions of the fuzz button are extracted to track the evolution of contact states between metal wires, as shown in Figure 6. By comparing the spatial distribution of metal wires at different loading stages, the meso-mechanical response mechanism of internal metal wires under compressive loading can be elucidated. Particularly in the crossing and bending regions of metal wires, the local normal contact pressure increases progressively with increasing compressive displacement. The contact modes evolve from point contact and local sliding in the initial stage to compressive contact and local sticking at larger compressive displacements, which further induces stress concentration and plastic accumulation.

To obtain the macroscopic mechanical response of the fuzz button, the displacement and reaction force data of the punch reference point were extracted throughout the loading–unloading process to construct the force–displacement hysteresis curve. This curve can directly characterize the load-bearing capacity, nonlinear stiffness variation, and hysteretic energy dissipation characteristics of the fuzz button. During data processing, the curve was divided into loading and unloading segments according to its variation trend, and baseline correction was performed on the original response. Nonphysical negative values near zero were truncated to zero to reduce the influence of numerical noise on the fitting results.

To eliminate the high-frequency numerical oscillations introduced by the explicit dynamic solution and extract the quasi-static response trend, a piecewise smoothing and fitting method was applied to the original curve. The loading and unloading segments were fitted using cubic smoothing splines, with smoothing factors of $S_{L}=3.50$ and $S_{U}=1.89$, respectively. The peak-displacement region was smoothed using the Savitzky–Golay method, with a window length of 9 and a polynomial order of 2. The fitting accuracy was quantitatively evaluated using the coefficient of determination $R^{2}$ and the root mean square error (RMSE). The residual was defined as $e_{i}=y_{i}-\hat{y_{i}}$, and the coefficient of determination was calculated as follows:

(9) $$R^{2}=1-\frac{\sum_{i=1}^{n}{\left(y_{i}-\hat{y_{i}}\right)}^{2}}{\sum_{i=1}^{n}{\left(y_{i}-\bar{y_{i}}\right)}^{2}}$$

The root mean square error was calculated as follows:

(10) $$RMSE=\sqrt{\frac{1}{n}\sum_{i=1}^{n}{\left(y_{i}-\hat{y_{i}}\right)}^{2}}$$

where $\hat{y_{i}}$ is the fitted value, $y_{i}$ is the original value, and $\bar{y_{i}}$ is the mean value of the original data.

**Figure 7:**
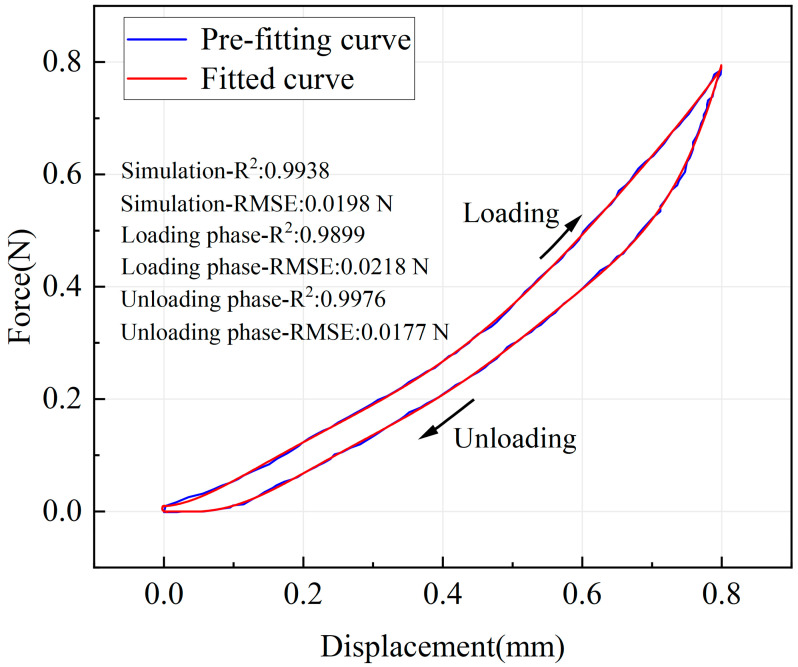
Comparison of simulation and fitted curve consistency.

Using Equations (9) and (10), the fitted curve was found to have $R^{2}=0.9938$ and RMSE= 0.0198 N. The comparison between the simulated and fitted curves is shown in Figure 7. The two curves exhibit good agreement, indicating that the fitting results accurately capture the variation characteristics of the loading–unloading curve.

Based on the force–displacement curve, the peak load and its corresponding displacement can be further extracted, and the average stiffness of the fuzz button during compression can be calculated [44]. The mechanical performance parameters of the mesoscopic fuzz button model are summarized in Table 13, where Δ*W* denotes the area enclosed by the hysteresis curve, *U* represents the maximum energy stored in the fuzz button during each cycle, *η* is the loss factor, defined as η=ΔW/(πU) and $K_{s}$ is the average stiffness of the fuzz button.

The virtual compression simulation reproduced the complete loading, holding, and unloading process. After elastic recovery, the final length of the fuzz button was 4.0322 mm, corresponding to a springback rate of approximately 0.805%. Assuming a constant wire volume and an unchanged diameter of 1.6 mm, the resulting filling ratio was 24.80%, which is close to the target value of 25%, as shown in Table 14.

## 4. Finite Element Simulation Validation

### 4.1. Experimental System and Testing Method

To validate the effectiveness of the established finite element simulation method, a typical compression condition was selected for comparative analysis between experimental testing and numerical simulation. The experimental specimens and finite element model were kept consistent in terms of geometric dimensions, material parameters, and loading displacement conditions to ensure the comparability of the results. A compressive strain of 20%, corresponding to a compression displacement of 0.8 mm, was selected as the validation condition. The force–displacement curve, peak load, stiffness characteristics, and hysteresis behavior were used as the primary evaluation metrics for a comprehensive comparison between the simulation and experimental results. The detailed preparation process parameters of the fuzz button specimens are listed in Table 15.

The comparison between experimental and simulation results enables validation of the rationality of the simulation method at both the macroscopic response and local feature levels. On the one hand, the overall mechanical curves of the fuzz button during the loading–unloading process were compared to verify the capability of the model to predict its nonlinear load-bearing behavior and rebound characteristics. On the other hand, the hysteresis area and residual deformation characteristics were analyzed to validate the ability of the model to characterize internal contact sliding and energy dissipation mechanisms.

**Figure 8:**
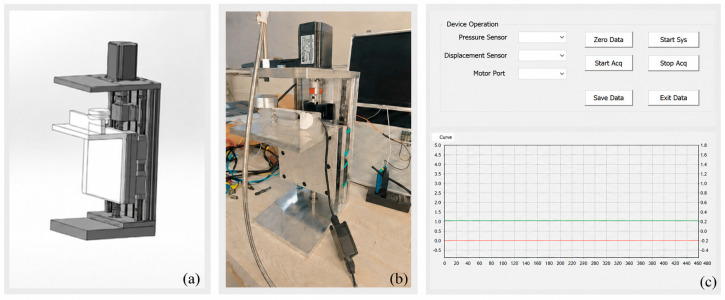
Fuzz button compression testing machine: (**a**) three-dimensional model; (**b**) physical photograph of the testing machine; (**c**) host-computer operating interface of the testing machine.

The experiments were conducted using a self-developed quasi-static compression testing machine, made by the School of Mechanical Engineering, University of Science and Technology Beijing, Beijing, China, as shown in Figure 8. The apparatus has a displacement resolution of 1.6 μm and a force resolution of 0.5 mN. The host-computer program provides functions for online recording, plotting, and data storage. The connection components were precision-machined from high-strength aluminum alloy and can satisfy the requirements for quasi-static compression testing of fuzz buttons with different dimensions within a small-deformation range.

During the experiment, the fuzz button specimen was placed between the upper and lower compression platens, and loading and unloading were performed under displacement control. 

### 4.2. Comparative Analysis of Simulation and Experimental Results

**Figure 9:**
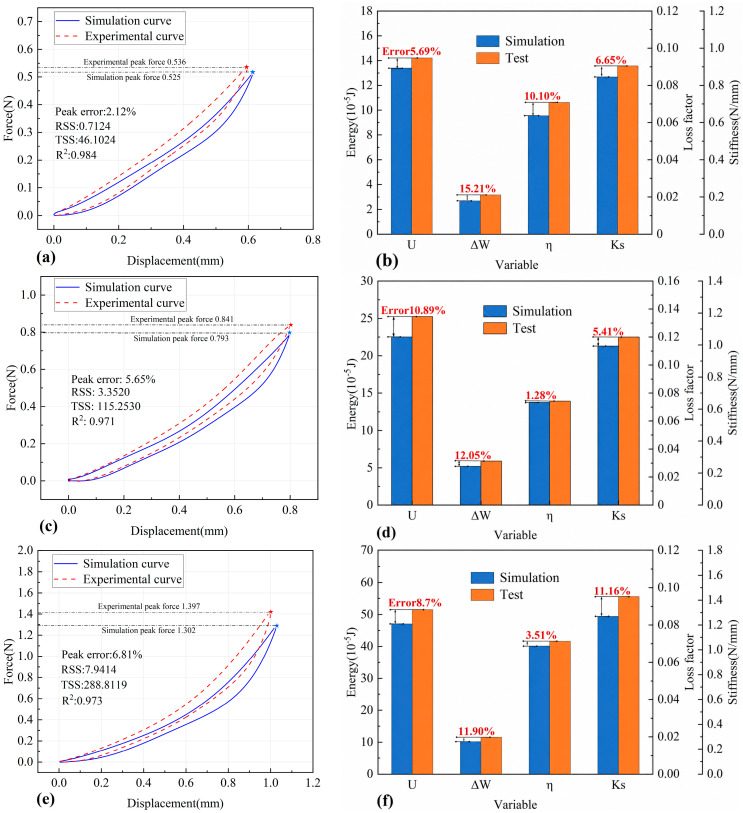
Experimental–numerical comparison of compressive hysteresis curves and characteristic parameters of fuzz buttons under different compressive strains: (**a**) force–displacement hysteresis curves at ε = 0.15; (**b**) characteristic parameters and relative errors at ε = 0.15; (**c**) force–displacement hysteresis curves at ε = 0.20; (**d**) characteristic parameters and relative errors at ε = 0.20; (**e**) force–displacement hysteresis curves at ε = 0.25; and (**f**) characteristic parameters and relative errors at ε = 0.25.

To further verify the predictive capability of the proposed finite element model, three compression levels were selected for comparison between numerical simulations and experiments. The compressive strains were 15%, 20% and 25%, corresponding to preset compressive displacements of 0.6 mm, 0.8 mm and 1.0 mm, respectively. For each compression condition, triplicate experiments were conducted to mitigate the effects of inter-sample variability caused by the randomness of internal metal wire arrangement. The experimental force–displacement curves used for comparison were obtained by averaging the results of the three repeated tests. The numerical predictions were then compared with the averaged experimental curves. For each loading condition, the simulated and experimental force–displacement curves were compared, and the peak reaction force, stored energy U, hysteresis energy ΔW, loss factor η and average stiffness K_S_ were extracted as evaluation indices. The results are presented in Figure 9.

Figure 9a shows the comparison between experimental and simulated curves under 15% compression. The simulated peak force is 0.5253 N and the experimental peak force is 0.5367 N, with a peak force error of 2.12%. The coefficient of determination R^2^ = 0.984, indicating excellent agreement between the simulated and experimental force–displacement curves. The relative errors of each mechanical index are shown in Figure 9b: 5.69% for stored energy U, 15.21% for hysteresis energy ΔW, 10.10% for loss factor η, and 6.65% for average stiffness K_s_. These results demonstrate that the model can accurately reproduce the compressive response at relatively low compression levels.

Figure 9c presents the comparison under 20% compression. The simulated peak force is 0.7936 N and the experimental peak force is 0.8411 N, with a relative error of 5.65%. The corresponding force–displacement curves also show good agreement with the experimental results, with R^2^ = 0.970. Figure 9d illustrates the relative errors of U, ΔW, η and K_s_, which are 10.89%, 12.05%, 1.28% and 5.41%, respectively. Compared with the 15% compression case, there is a slight increase in the deviations of stored energy and hysteresis energy, which can be attributed to the enhanced nonlinear contact evolution and more pronounced relative sliding between metal wires under larger deformations. The comparison of experimental and simulated curves for fuzz buttons under 25% compression is shown in Figure 9e. It can be seen that the simulated peak force is 1.3021 N and the experimental peak force is 1.3973 N, with a peak force error of 6.81%. The coefficient of determination remains at a high level (R^2^ = 0.973), indicating that the overall nonlinear trend of the force–displacement response is still well captured. Figure 9f shows the relative errors of U, ΔW, η and K_s_, which are 8.70%, 11.90%, 3.51% and 11.16%, respectively. Although the error in average stiffness increases under this larger compression condition, the model still reasonably predicts the load-bearing capacity, hysteretic response and energy dissipation characteristics of the fuzz button.

The validation results under 15%, 20% and 25% compression conditions show that the peak force errors remain within 7% in all three cases, and the force–displacement curves exhibit good agreement with the experimental results. The deviations observed at higher compression levels mainly originate from the randomness of internal metal wire arrangement, evolution of contact states, local sliding and stick–slip transitions, and possible inter- specimen variability. This indicates that the established model can effectively reflect the macroscopic energy dissipation behavior of fuzz buttons caused by relative sliding and frictional energy dissipation between internal metal wires, providing a reliable basis for subsequent design optimization and performance prediction.

Although the simulation and experimental results exhibit good agreement in key indicators, including the force–displacement response, peak load, stiffness evolution, and hysteresis characteristics, slight deviations remain between them. The main reasons are as follows: the winding configuration of metallic wires inside the real fuzz button exhibits pronounced randomness, and the virtual preparation model cannot fully reproduce the specific initial state and pre-contact conditions of each wire; the initial residual stress, local defects, and material property dispersion that may exist in the actual specimens were reasonably simplified in the model; and the adopted contact-related parameters may also deviate slightly from their true values. The errors caused by these factors are within the acceptable range for engineering applications and do not affect the validity of the model or its subsequent application.

## 5. Conclusions

To address the complex mesostructure, pronounced contact nonlinearity, and difficulty in accurately characterizing the compressive mechanical behavior of fuzz button contacts, a mesoscopic finite element model of the fuzz button based on virtual fabrication was established in this study. The validity of the model was further verified by quasi-static compression experiments. The main conclusions are as follows:

(1) A parametric method for generating the spatial trajectories of metallic wires was proposed based on the actual fabrication process and three-dimensional braiding concept. After trajectory smoothing and virtual compression forming in Abaqus, a mesoscopic finite element model with realistic geometrical morphology and internal contact topology was obtained.
(2) A complete finite element simulation procedure was developed, including mesh generation, contact definition, analysis step configuration, boundary condition assignment, and post-processing. The model successfully reproduced the nonlinear stiffness enhancement and hysteretic energy dissipation behavior of the fuzz button during the loading–unloading process.
(3) The model was validated at compressive strains of 15%, 20%, and 25%, corresponding to compression displacements of 0.6, 0.8, and 1.0 mm, respectively. The relative errors in the predicted peak forces were 2.12%, 5.65%, and 6.81%, while the corresponding coefficients of determination were 0.984, 0.970, and 0.973. The maximum relative error among the evaluated energy- and stiffness-related indicators was 15.21%. These results demonstrate that the proposed model can reliably reproduce the nonlinear force–displacement response and hysteretic energy dissipation of fuzz buttons over the investigated compression range.
(4) By incorporating the virtual manufacturing sequence from wire-trajectory generation to compression densification, the proposed framework establishes a direct relationship among mesoscopic geometry, contact evolution, and macroscopic mechanical response. It enables the simultaneous investigation of force–displacement behavior, local stress concentration, and hysteretic energy dissipation, which are difficult to resolve using purely homogenized descriptions. A quantitative comparison of computational efficiency and prediction accuracy with homogenized and spring-network models will be conducted in future work.

## Figures and Tables

**Table 1:** Four-Step Method–Periodic Braiding Steps.

Step	Direction of Motion	Even Rows/Columns	Odd Rows/Columns	Z Direction
1	Along X direction	(x − 1, y, z + 1)—move left	(x + 1, y, z + 1)—move right	Z + 1
2	Along Y direction	(x, y − 1, z + 1)—move up	(x, y + 1, z + 1)—move down	Z + 1
3	Along X direction (reverse)	(x + 1, y, z + 1)—move right	(x − 1, y, z + 1)—move left	Z + 1
4	Along Y direction (reverse)	(x, y + 1, z + 1)—move down	(x, y − 1, z + 1)—move up	Z + 1

**Table 2:** Parameters of the fuzz button after forming.

Diameter (mm)	Height (mm)	Wire Diameter (mm)	Filling Ratio (%)	Porosity (%)
1.6	4	0.08	25	75

**Table 3:** Optimal pairing parameters of the preform.

Name	Core Coding Region Size	Motion Step Length *A* (mm)	Motion Step Length *B* (mm)	Reset Times $N_{reset}$
Parameter value	4	0.246	0.540	1

**Table 4:** Specification parameters of the fuzz button preform.

Name	Geometric Outline Side Length (mm)	Height (mm)	Wire Diameter (mm)	Volume (mm^3^)
Fuzz button	1.31	12.96	0.08	2.0106

**Table 5:** Material Property Settings.

Density (g/cm^3^)	Young’s Modulus (GPa)	Poisson’s Ratio	Bulk Modulus (GPa)	Shear Modulus (GPa)
8.25	128	0.3	106.7	49.2

**Table 6:** Comparison of simulation results with and without Nix–Gao correction.

Model	Peak Reaction Force (N)	Peak Force Error (%)	*R* ^2^
Without Nix–Gao correction	0.7798	7.29	0.965
With Nix–Gao correction	0.7936	5.65	0.970
Experimental result	0.8411	/	/

**Table 7:** Mesh-convergence analysis of the fuzz button model.

Mesh Case	Global Mesh Size (mm)	Number of Elements	Peak Force (N)	Relative Error (%)
M1	0.080	19,200	0.8415	4.48
M2	0.065	30,053	0.8340	3.55
M3	0.052	60,108	0.7936	1.47
M4	0.040	137,046	0.8054	0

**Table 8:** Number and types of elements for each component.

Item	Braided Button Preform	Punch	Die
Total number of elements	60,108	3520	15,997
Element type	C3D8R	C3D8R	C3D10M
Approximate global size/mm	0.052	0.25	0.25
Maximum deviation factor	0.1	0.1	0.1
Element order	First order	First order	First order
Mesh type	Hexahedron	Hexahedron	Tetrahedron

**Table 9:** Penetration for different initial contact stiffness values.

**Penalty stiffness scale factor**	0.2	0.6	0.8	1	5
**Penetration (mm)**	0.12	0.0034	0.0026	0.0013	Non-convergence

**Table 10:** Average dissipated energy and energy dissipation ratio under different friction coefficients.

*μ*	${\bar{W}}_{load}$ (10^−5^ J)	${\bar{W}}_{d}$ (10^−5^ J)	$\bar{\lambda }$ (%)	Average Peak Force (N)
0.10	24.51	5.1177	20.88	0.7993
0.12	24.58	5.1547	20.96	0.7783
0.15	23.87	5.5122	23.07	0.7666
0.20	23.53	6.3383	26.88	0.7175
0.25	22.27	6.1742	27.60	0.7013

**Table 11:** Contact Property Settings.

Contact Property	Friction Coefficient	Pressure Interference	Friction Formulation
Tangential Behavior	0.12	/	Penalty function
Normal Behavior	/	Hard contact	/

**Table 12:** Analysis Settings.

Analysis Step	Geometric Nonlinearity	Time Period	Natural Frequency	Increment Step
Loading	Yes	1	100	1 × 10^−5^
Unloading	Yes	1	100	1 × 10^−5^

**Table 13:** Mechanical properties of the meso-model of the fuzz button.

Δ*W* (10^−5^ J)	*U* (10^−5^ J)	*H*	*K_S_* (N/mm)
5.1803	22.5002	0.07329	0.9938

**Table 14:** Analysis of Compression Molding Parameters for Fuzz Buttons.

Parameter Item	Length (mm)	Diameter (mm)	Filling Ratio (%)
Target Parameters	4.00	1.60	25
Actual Parameters	4.03	1.60	24.80

**Table 15:** Specifications of the fuzz button.

Diameter (mm)	Height (mm)	Wire Diameter (mm)	Filling Ratio (%)	Porosity (%)
1.6	4	0.08	25	75

## Data Availability

The original contributions presented in this study are included in the article. Further inquiries can be directed to the corresponding author.
